# Reply: Crossover Handling and Atrial Fibrillation Burden in PRAGUE-25

**DOI:** 10.1016/j.jacadv.2026.103418

**Published:** 2026-11-03

**Authors:** Zuzana Hejdukova, Marek Hozman, Pavel Osmancik

**Affiliations:** Cardiocenter, Department of Cardiology, Third Faculty of Medicine, Charles University, University Hospital Kralovske Vinohrady, Prague, Czech Republic

We thank Dr Nasir for highlighting the important issue of crossover handling in relation to the *per-protocol* (PP) analysis of PRAGUE-25.^1^ We agree that intention-to-treat (ITT) and PP analyses address different estimands. Importantly, in PRAGUE-25, both are particularly relevant because the trial was designed as a *noninferiority* trial. In *noninferiority* trials, crossover may make treatment groups more similar and thereby bias ITT analyses toward noninferiority. Accordingly, both ITT and PP analyses are recommended, and both were prespecified in PRAGUE-25.^2^ The present PP analysis was therefore intended as a complementary analysis, not as a data-driven replacement of the original ITT analysis.^1^

The treatment-policy ITT analysis of atrial fibrillation (AF) burden at 12 months, as suggested by Dr Nasir, was already reported in the primary trial (12.1 ± 31.2% with catheter ablation [CA] vs 22.1 ± 37.2% with lifestyle modification plus antiarrhythmic drugs [LFM + AAD], *P* = 0.17), but we agree that corresponding analyses at 6 and 9 months were not reported.^2^

Importantly, 14 patients crossed from LFM + AAD to CA during the first year, compared with only 1 crossover in the opposite direction. Thus, postcrossover Holter recordings assigned to the original LFM + AAD group partly reflected the effect of CA.

As suggested, we also examined AF burden before crossover. A protocol-scheduled Holter was available before crossover in 12 of 14 patients. The median interval between the last Holter and crossover was 43.5 days (IQR: 30.0-53.8). At 6 months, AF burden did not differ between patients who subsequently crossed over and those remaining on LFM + AAD (7.53% [IQR: 0%-34.4%] vs 0.49% [IQR: 0%-63.2%]; *P* = 0.77). At 9 months, however, AF burden was higher in patients who subsequently crossed over (61.4% [18.3%-100%] vs 0% [0%-27.3%]; *P* = 0.021). Thus, censoring crossovers removed patients with poorer rhythm control.

In addition, we repeated the AF-burden analysis using a treatment-policy ITT estimand, retaining patients according to randomized allocation irrespective of crossover. The results are presented as mean ± SD to maintain consistency with the primary publication, in which 12-month AF burden was also reported using mean ± SD. The mean AF burden was 9.3% ± 25.7% in the CA group vs 30.1% ± 41.7% in the LFM + AAD group (*P* = 0.006), and 10.3% ± 28.9% vs 21.9% ± 38.1% (*P* = 0.288), at 6 and 9 months, respectively. The direction of the treatment effect on AF burden was nevertheless consistent between ITT and PP analyses, as well as with the results of the primary endpoint. But as can be seen, the between-group differences were attenuated in the ITT analysis, with crossover accumulating over time during follow-up.

Finally, missing Holter data were infrequent—approximately 3% at each milestone—and are unlikely to explain the findings. Taken together, the ITT and PP analyses provide complementary evidence, with both pointing in the same direction. It also emphasizes the need for PP analysis as a standard part of *noninferiority* trials.
